# Sensitization of ASIC3 by proteinase-activated receptor 2 signaling contributes to acidosis-induced nociception

**DOI:** 10.1186/s12974-017-0916-4

**Published:** 2017-07-28

**Authors:** Jing Wu, Ting-Ting Liu, Yi-Mei Zhou, Chun-Yu Qiu, Ping Ren, Ming Jiao, Wang-Ping Hu

**Affiliations:** 1 Research Center of Basic Medical Sciences, School of Basic Medical Sciences, Hubei University of Science and Technology, 88 Xianning Road, Xianning, 437100 Hubei People’s Republic of China; 2 Department of Physiology, School of Basic Medical Sciences, Hubei University of Science and Technology, 88 Xianning Road, Xianning, 437100 Hubei People’s Republic of China; 3 Department of Pharmacology, Hubei University of Science and Technology, 88 Xianning Road, Xianning, 437100 Hubei People’s Republic of China

**Keywords:** Proteinase-activated receptor 2, Acid-sensing ion channel 3, Proton-gated current, Nociception, Dorsal root ganglion neuron

## Abstract

**Background:**

Tissue acidosis and inflammatory mediators play critical roles in pain. Pro-inflammatory agents trypsin and tryptase cleave and activate proteinase-activated receptor 2 (PAR_2_) expressed on sensory nerves, which is involved in peripheral mechanisms of inflammation and pain. Extracellular acidosis activates acid-sensing ion channel 3 (ASIC3) to trigger pain sensation. Here, we show that a functional interaction of PAR_2_ and ASIC3 could contribute to acidosis-induced nociception.

**Methods:**

Electrophysiological experiments were performed on both rat DRG neurons and Chinese hamster ovary (CHO) cells expressing ASIC3 and PAR_2_. Nociceptive behavior was induced by acetic acid in rats.

**Results:**

PAR_2_-AP, PAR_2_-activating peptide, concentration-dependently increased the ASIC3 currents in CHO cells transfected with ASIC3 and PAR_2_. The proton concentration–response relationship was not changed, but that the maximal response increased 58.7 ± 3.8% after pretreatment of PAR_2_-AP. PAR_2_ mediated the potentiation of ASIC3 currents via an intracellular cascade. PAR_2_-AP potentiation of ASIC3 currents disappeared after inhibition of intracellular G protein, PLC, PKC, or PKA signaling. Moreover, PAR_2_ activation increased proton-evoked currents and spikes mediated by ASIC3 in rat dorsal root ganglion neurons. Finally, peripheral administration of PAR_2_-AP dose-dependently exacerbated acidosis-induced nocifensive behaviors in rats.

**Conclusions:**

These results indicated that PAR_2_ signaling sensitized ASIC3, which may contribute to acidosis-induced nociception. These represent a novel peripheral mechanism underlying PAR_2_ involvement in hyperalgesia by sensitizing ASIC3 in primary sensory neurons.

## Background

Proteinase-activated receptors (PARs) are a subfamily of G protein-coupled receptors (GPCRs) and have at least four members (PAR_1_, PAR_2_, PAR_3_, and PAR_4_) [1]. PARs can be activated by several proteases such as trypsin and tryptase, which are generated during tissue injury and inflammation [2]. These proteases cleave PARs at a specific site within the extracellular amino-terminus and subsequently expose amino-terminal to the tethered ligand domain that binds to and activates the cleaved receptors [3]. Some specific synthetic peptides have been shown to activate the specific PARs and mimic the effects of the activating proteases. For example, 2-furoyl-LIGRLO-NH2 and SLIGKV-NH2 are used for PAR_2_ activation [4]. Once activated, PARs regulate multiple pathophysiological processes including inflammation, pain, hemostasis, and healing [2, 5, 6]. It has been found that PAR_2_ is expressed on a subset of primary sensory neurons and functionally involved in peripheral mechanisms of inflammation and pain [7, 8]. PAR_2_ is co-localized with substance P and calcitonin gene-related peptide in over 60% of dorsal root ganglion (DRG) neurons, and activation of PAR_2_ on sensory nerve ending evokes the release of these peptides in peripheral tissues [7]. Intraplantar injection of subinflammatory doses of PAR_2_ agonists in rats and mice induces thermal and mechanical hyperalgesia [9]. PAR_2_-deficient mice fail to show nociceptive sensitization in many inflammatory pain models [9]. In addition, PAR_2_ is found to play an important role in postoperative, neuropathic, and cancer pain [6, 10–12]. PAR_2_ signaling is sufficient to induce the transition to a chronic pain state [13]. It is reported that PAR_2_ activation can sensitize rat DRG neurons in vitro and may contribute to the pathogenesis of pain [7, 8]. PAR_2_ activation leads also to sensitization of transient receptor potential (TRP) channels, including TRPV1, TRPV4, and TRPA1, which are crucial for nociceptive signaling and modulation. It has been demonstrated that thermal hyperalgesia induced by intraplantar injection of PAR_2_ agonist is dependent on TRPV1 activation [10, 14, 15]. Mechanical hyperalgesia evoked by peripheral activation of PAR_2_ is prevented in TRPV4 knock-out mice [16, 17]. Sensitization of TRPA1 by PAR_2_ activation contributes to inflammatory pain and paclitaxel-induced mechanical, heat, and cold hypersensitivity [10, 18]. Thus, TRPV1, TRPV4, and TRPA1 mediate the pronociceptive actions of PAR_2_.

Acid-sensing ion channels (ASICs) are proton-gated cation channels which are activated by extracellular pH fall. To date, at least six ASIC subunits encoded by four genes have been identified in mammals [19]. Most of the ASIC subunits (i.e., ASIC1a and b, ASIC2a and b, and ASIC3) are expressed in both DRG cell bodies and sensory terminals, which contribute to proton-evoked pain signaling [20–22]. It has been demonstrated that application of an acidic solution into the skin depolarizes the terminals of nociceptive primary sensory neurons to cause pain sensation by activating ASICs, rather than TRPV1 [21, 23]. Among the ASIC subunits, ASIC3 displays higher sensitivity to extracellular protons than other ASICs, with activation thresholds just below the physiological pH value (around pH 7.2) [24]. During inflammation, tissue injury, ischemic stroke, and surgical trauma, proton is released and decreases extracellular pH level [25]. The released proton is sufficient to activate ASIC3 and can trigger pain sensation [26]. ASIC3 is specifically localized in nociceptive fibers innervating the skeletal and cardiac muscles, joints, and bone [27, 28]. Activation of ASIC3 in sensory neurons has been proposed to contribute to the generation of pain. Blocking ASIC3 at the periphery inhibits the spontaneous pain generated by mild cutaneous acidification, reverses CFA-induced primary hyperalgesia, and reduces post-operative pain behaviors when applied to the incised area during surgery [21, 29, 30]. Increasing evidence has shown that ASIC3 plays an important role in various pain conditions such as inflammatory pain, postoperative pain, and migraine [22, 29, 31].

We report here a functional interaction between PAR_2_ and ASIC3 in both rat DRG neurons and Chinese hamster ovary (CHO) cells expressing ASIC3 and PAR_2_, which contributes to acidosis-induced nociception in rats.

## Methods

### Cell culture and transfection

ASIC3, ASIC1a, ASIC1b, ASIC2b, and PAR_2_ complementary DNAs (cDNAs) were used for heterologous expression in CHO cells as described previously (Wang et al., 2013). In brief, CHO cells were cultured at 37 °C in a humidified atmosphere of 5% CO_2_ and 95% O_2_ and passaged twice a week. Transient transfection of CHO cells was performed using HilyMax liposome transfection reagent (Dojindo Laboratories). CHO cells were maintained in F-12 Nutrient Mixture (added 1.176 g of NaHCO_3_/L medium) supplemented with 10% fetal bovine serum and 1% gluta-MAXTM-1 (100×; Invitrogen). When ASIC3 and PAR_2_ cDNAs were co-transfected, the ratio was kept at 1:1. All plasmids used contained, in addition to the desired ASIC3 cDNA, the coding sequence for enhanced green fluorescent protein to aid identification of transfected cells. Electrophysiological measurements were performed 24–48 h after transfection.

### Isolation of DRG neurons

The experimental protocol was approved by the animal research ethics committee of Hubei University of Science and Technology (No. 2016–67). All procedures conformed to international guidelines on the ethical use of animals, and every effort was made to minimize the number of animals used and their sufferings. Five- to 6-week-old Sprague–Dawley male rats were anesthetized with 7% chloral hydrate and then decapitated. The DRGs were taken out and transferred immediately into Dulbecco’s modified Eagle’s medium (DMEM, Sigma) at pH 7.4. After the removal of the surrounding connective tissues, the DRGs were minced with fine spring scissors and the ganglion fragments were placed in a flask containing 5 ml of DMEM in which trypsin (type II-S, Sigma) 0.5 mg/ml, collagenase (type I-A, Sigma) 1.0 mg/ml, and DNase (type IV, Sigma) 0.1 mg/ml had been dissolved and incubated at 35 °C in a shaking water bath for 25–30 min. Soybean trypsin inhibitor (type II-S, Sigma) 1.25 mg/ml was then added to stop trypsin digestion. The incubating solution was then replaced by external solution. Dissociated neurons were placed into a 35-mm Petri dish and kept for at least 1 h in normal external solution before the start of electrophysiological experiments. After plating of the DRG neurons, the neurons were used for experiments within 24 h. The neurons selected for electrophysiological experiment were 15–35 μm in diameter.

### Electrophysiological recordings

Whole-cell patch clamp and voltage clamp recordings were carried out at room temperature (22–25 °C) using a MultiClamp-700B amplifier and Digidata-1440A A/D converter (Axon Instruments, CA, USA). Recording pipettes were pulled using a Sutter P-97 puller (Sutter Instruments, CA, USA). The micropipettes were filled with internal solution containing (mM) KCl 140, MgCl_2_ 2.5, HEPES 10, EGTA 11, and ATP 5; its pH was adjusted to 7.2 with KOH, and osmolarity was adjusted to 310 mOsm/L with sucrose. Cells were bathed in an external solution containing (mM) NaCl 150, KCl 5, CaCl_2_ 2.5, MgCl_2_ 2, HEPES 10, d-glucose 10; its osmolarity was adjusted to 330 mOsm/L with sucrose and its pH to 7.4. The resistance of the recording pipette was in the range of 3–6 MΩ. A small patch of membrane underneath the tip of the pipette was aspirated to form a giga seal, and then, a negative pressure was applied to rupture it, thus establishing a whole-cell configuration. The series resistance was compensated for by 70–80%. The adjustment of capacitance compensation was also done before recording the membrane currents. The membrane voltage was maintained at −60 mV in all voltage clamp experiments unless otherwise specified. Current clamp recordings were obtained by switching to current clamp mode after a stable whole-cell configuration was formed in voltage clamp mode. Only cells with a stable resting membrane potential (more negative than −50 mV) were used in the study. Signals were sampled at 10 to 50 kHz and filtered at 2 to 10 kHz, and the data were stored in compatible PC computer for off-online analysis using the pCLAMP 10 acquisition software (Axon Instruments, CA, USA).

### Drug application

Drugs purchased from Sigma and used in the experiments include hydrochloric acid, 2-furoyl-LIGRLO-NH2 (a PAR_2_-activating peptide (PAR_2_-AP)), trypsin, FSLLRY-NH2, APETx2, and capsazepine. Different pH values were configured with hydrochloric acid and external solution. All drugs were dissolved daily in the external solution just before use and held in a linear array of fused silica tubes (o.d./i.d. = 500 μm/200 μm) connected to a series of independent reservoirs. The application pipette tips were positioned ∼30 μm away from the recorded neurons. The application of each drug was driven by gravity and controlled by the corresponding valve, and rapid solution exchange could be achieved within about 100 ms by shifting the tubes horizontally with a PC-controlled micromanipulator. Cells were constantly bathed in normal external solution flowing from one tube connected to a larger reservoir between drug applications. In some experiments where GDP-β-S (Sigma), U-73122(Sigma), and GF109203X (RBI) were applied for intracellular dialysis through recording patch pipettes, they were dissolved in the internal solution before use. To ensure that the cell interior was perfused with the dialysis drug, there was at least a 30-min interval between the establishment of whole-cell access and the current measurement.

### Nociceptive behavior induced by acetic acid in rats

Rats were placed in a 30 × 30 × 30 cm Plexiglas chamber and allowed to habituate for at least 30 min before nociceptive behavior experiments. A blind experiment was carried out. Separate groups of rats were coded and pretreated with 20 μl capsazepine (100 μM) together with vehicle and different dosages of PAR_2_-AP, FSLLRY-NH2, or APETx2 in the ipsilateral hind paw before injection of acetic acid. After 5 min, the other experimenters who did not know the above experimental condition subcutaneously administered acetic acid solution (0.6%, 20 μl) into the dorsal face of the hind paw using a 30-gauge needle connected to a 100-μl Hamilton syringe. And nociceptive behavior (that is, number of flinches) was counted over a 5-min period starting immediately after the injection [21, 32].

### Data analysis

Data were statistically compared using the Student’s *t* test or analysis of variance (ANOVA), followed by Bonferroni’s post hoc test. Statistical analysis of concentration–response data was performed using nonlinear curve-fitting program ALLFIT. Data are expressed as mean ± SEM.

## Results

### Enhancement of proton-gated currents by PAR_2_ agonist in CHO cells co-expressing ASIC3 and PAR_2_

**Fig. 1:**
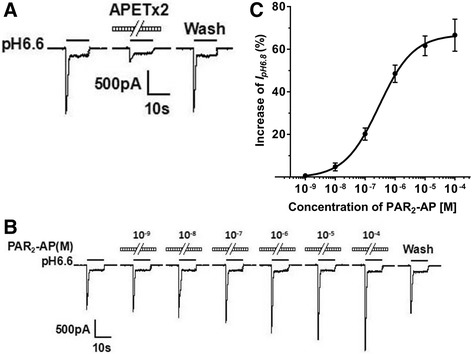
Potentiation of proton-gated currents by PAR_2_-AP in CHO cells co-expressing ASIC3 and PAR_2_. **a** Representative traces show currents evoked by a pH 6.6 acidic solution for 5 s in CHO cells co-expressing ASIC3 and PAR_2_. The proton-gated current could be blocked by 500 nM APETx2, an ASIC3 inhibitor. **b** The sequential current traces illustrate the potentiation of proton-gated currents by different concentrations of PAR_2_-activating peptide (PAR_2_-AP: 2-furoyl-LIGRLO-NH2, 10^−9^–10^−4^ M). Representative currents were recorded for more than 60 min in a cell with membrane potential clamped at −60 mV. PAR_2_-AP was pre-applied to external solution for 1 min. **c** The graph shows PAR_2_-AP increased the peak amplitude of proton-gated currents in a concentration-dependent manner with an EC_50_ of 2.9 × 10^−7^ M. Each point represents the mean ± SEM of 8 to 10 cells

To investigate the functional interaction of the ASIC3 with PAR_2_, ASIC3 and PAR_2_ cDNAs were co-transfected into CHO cells in the present study. We first examined the effects of a PAR_2_-activating peptide (PAR_2_-AP: 2-furoyl-LIGRLO-NH2) on the proton-gated currents in CHO cells co-expressing ASIC3 and PAR_2_ using a whole-cell patch clamp technique. A rapid reduction of extracellular pH from 7.4 to 6.6 for 5 s evoked an inward current (*I*_pH 6.6_) in CHO cells transfected with ASIC3 and PAR_2_ under the voltage clamp conditions. These acidosis-evoked currents were characterized by a large transient peak current followed by fast inactivation and then a small sustained current with no or very slow inactivation (Fig. 1a) [33]. APETx2 (500 nM), an ASIC3 blocker, inhibited the peak ASIC current without affecting the sustained plateau; thus, they may be considered to be ASIC3 currents (Fig. 1a). In addition, a pH 6.6 acidic stimulus did not induce any significant current in untransfected CHO cells (data not shown).

We observed that transient peak ASIC3 currents were enhanced by the pre-application of PAR_2_-AP for 1 min (Fig. 1b, c). And the potentiation of transient peak ASIC3 currents was dependent upon the concentration of PAR_2_-AP. Figure 1b shows that the peak amplitude of *I*_pH 6.6_ increased as concentration of pre-treated PAR_2_-AP increased from 10^−9^ to 10^−4^ M in a representative CHO cell co-expressing ASIC3 and PAR_2_. The enhancing effect of PAR_2_-AP was reversible in washout experiments. Figure 1c shows the concentration–response curve for PAR_2_-AP in the potentiation of ASIC3 currents. PAR_2_-AP had a maximum effect (66.6 ± 7.5%, *n* = 9) at a concentration of 10^−4^ M. The half-maximal response (EC_50_) value and Hill coefficient of the concentration–response curve for PAR_2_-AP were (2.9 ± 0.2) × 10^−7^ M and 0.76, respectively. The results indicated that PAR_2_-AP enhanced the ASIC3 currents in a concentration-dependent manner.

### Activation and steady-state desensitization of ASIC3 expressed in CHO cells with and without pretreatment of PAR_2_-AP

**Fig. 2:**
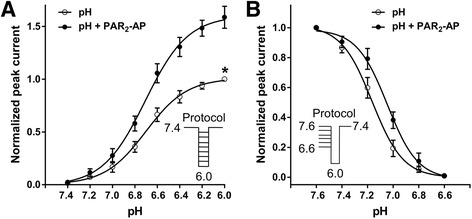
Concentration–response relationship for protons and steady-state desensitization of ASIC3 with or without the pre-application of PAR_2_-AP. **a** The concentration–response curves for protons with or without 10^−5^ M PAR_2_-AP pre-application in CHO cells co-expressing ASIC3 and PAR_2_. Each point represents the mean ± SEM of 8 to 10 neurons. All current values were normalized to the current response induced by pH 6.0 applied alone (marked with *asterisk*). The curves shown are a best fit of the data to the logistic equation *I* = *I*_max_/[1 + (pH_50_/pH)^*n*^], where pH is the pH value used, *I* is the normalized current response value, pH_50_ is the pH value for half-maximal current response, and *n* is the Hill coefficient. The curves for protons without and with PAR_2_-AP pre-application were drawn according to the equation described above. **b** Steady-state desensitization of homomeric ASIC3 expressed in CHO cells with or without PAR_2_-AP pre-application. PAR_2_-AP (10^−5^ M) induced a rightward shift of the pH dependence of steady-state desensitization. Each point represents the mean ± SEM of 6 to 8 neurons. The holding pH varied from 7.6 to 6.6. All currents were induced by pH 6.0 applied alone

We then investigated whether the potentiation of ASIC3 currents by PAR_2_-AP was dependent upon pHs. Figure 2a shows the concentration–response curves to protons in the presence and absence of PAR_2_-AP (10^−5^ M). First, after pretreatment of PAR_2_-AP, the proton concentration–response relationship was not changed, but that, the maximal response increased, as indicated by an increase of 58.7 ± 3.8% in the maximal current response to protons when PAR_2_-AP was pre-applied. However, the slopes or Hill coefficients of those two curves were essentially similar (*n* = 2.36 ± 0.13 in the absence of PAR_2_-AP versus *n* = 2.28 ± 0.15 in the presence of PAR_2_-AP; *P* > 0.1, Bonferroni’s post hoc test). Second, the pH values for half-maximal current response (pH_50_) of both curves had no statistical difference (pH_50_ of 6.70 ± 0.02 without PAR_2_-AP pretreatment versus pH_50_ of 6.71 ± 0.04 with PAR_2_-AP pretreatment; *P* > 0.1, Bonferroni’s post hoc test). Third, the threshold pH values of both curves had no significant difference in the presence and absence of PAR_2_-AP.

Next, we compared the desensitization properties of ASIC3 currents in the absence or presence of PAR_2_-AP. Steady-state desensitization was examined by superfusion of CHO cells co-expressing ASIC3 and PAR_2_ for 2 min in solutions with pH values ranging from 7.6 to 6.6 before application of the pH 6.0 solution. PAR_2_-AP (10^−5^ M) induced a rightward shift of the pH dependence of steady-state desensitization. The pH_50_ value for steady-state desensitization changed from 7.16 ± 0.01 to 7.05 ± 0.02 with the presence of 10^−5^ M PAR_2_-AP (*P* < 0.05, Bonferroni’s post hoc test; Fig. 2b), indicative of a decreased apparent proton affinity under steady-state conditions. The Hill coefficients were 3.58 ± 0.29 without PAR_2_-AP and 3.65 ± 0.35 with PAR_2_-AP.

### The receptor and intracellular signal transduction mechanisms underlying potentiation of ASIC3 currents by PAR_2_-AP

**Fig. 3:**
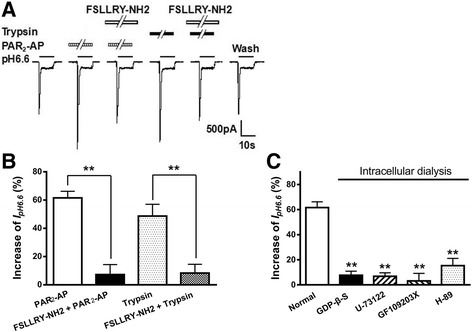
The receptor and intracellular mechanisms underlying the potentiation of ASIC3 currents by the activation of PAR_2_. The **a** current traces and **b** bar graphs show that *I*_pH 6.6_ was enhanced by PAR_2_-AP (10^−5^ M) pre-applied alone for 1 min in CHO cells co-expressing ASIC3 and PAR_2_. This enhancing effect was inhibited by the co-application of PAR_2_-AP and FSLLRY-NH2 (10^−5^ M), a selective PAR_2_ antagonist. Trypsin, another PAR_2_ agonist, has a similar increasing effect on *I*_pH 6.6_ at concentration of 10^−5^ M for 1 min. And the enhancing effect of trypsin was also inhibited by FSLLRY-NH2 (10^−5^ M). Statistical tests were performed using Bonferroni’s post hoc test, and significance is shown as follows: **P < 0.01, compared with *white column. n* = 10 in each column. The **c** bar graph shows the percentage increases in the *I*_pH 6.6_ induced by PAR_2_-AP (10^−5^ M) with recording pipettes filled with the normal internal solution, non-hydrolyzable GDP analog GDP-β-S (500 μM), PLC inhibitor U-73122 (10 μM), PKC inhibitor GF109203X (2 μM), or H-89 (10 μM) containing internal solution. Intracellular dialysis of GDP-β-S, U-73122, GF109203X, or H-89 abolished the enhancing effect of PAR_2_-AP on *I*_pH 6.6_. **P < 0.01, post hoc Bonferroni’s test, compared with normal internal solution. *n* = 10 in each column

To verify whether the PAR_2_-AP potentiation of ASIC3 currents was mediated by PAR_2_, we co-applied PAR_2_-AP with FSLLRY-NH2, a selective PAR_2_ antagonist. The peak amplitude of *I*_pH 6.6_ increased 61.6 ± 4.6% after pretreatment with PAR_2_-AP (10^−5^ M) alone for 1 min in ten CHO cells co-expressing ASIC3 and PAR_2_ (Fig. 3a, b). In contrast, PAR_2_-AP produced an increase of 7.3 ± 7.1% on ASIC3 currents in ten cells pretreated with 10^−5^ M FSLLRY-NH2. And the peak amplitude of *I*_pH 6.6_ changed within 5% after pretreatment with 10^−5^ M FSLLRY-NH2 alone. Thus, the potentiation of *I*_pH 6.6_ by pretreatment with PAR_2_-AP could be blocked by the addition of FSLLRY-NH2 (one-way analysis of variance followed by post hoc Bonferroni’s test, *P* < 0.01, *n* = 10; Fig. 3a, b). As a possible physiological ligand of the PAR_2_, trypsin can cleave PAR_2_ within the extracellular N-terminal domains and then activate the cleaved receptor [3]. Similar to PAR_2_-AP, pretreatment of 10^−5^ M trypsin for 1 min also caused an increase of 48.7 ± 8.3% on ASIC3 currents in ten CHO cells co-expressing ASIC3 and PAR_2_ (Fig. 3a, b). The enhancing effect of trypsin was also inhibited by FSLLRY-NH2. And trypsin produced an increase of 8.4 ± 6.2% on ASIC3 currents in ten cells pretreated with 10^−5^ M FSLLRY-NH2 (Fig. 3a, b).

We further explored the signaling pathway in the downstream of PAR_2_ for sensitization of ASIC3. We recently reported that G_q/11_-coupled metabotropic receptor activation such as glutamate (mGluRs), ATP (P2Y), and serotonin (5-HT_2_) receptors causes potentiation of ASICs in a PKC-dependent manner in rat DRG neurons [34–36]. Therefore, we examined whether a similar signal transduction pathway is involved in the modulation of ASIC3 by the activation of PAR_2_, a member of the G_q/11_-coupled metabotropic receptor family. GDP-β-S (a non-hydrolyzable GDP analog, 500 μM), U-73122 (a PLC inhibitor, 10 μM), or GF109203X (a selective PKC inhibitor, 2 μM) was applied internally to CHO cells through recording patch pipettes. As shown in Fig. 3c, pre-application of PAR_2_-AP (10^−5^ M for 1 min) increased *I*_pH 6.6_ to 7.7 ± 3.2, 6.9 ± 2.8, and 3.2 ± 6.0%, separately, when GDP-β-S, U-73122, or GF109203X was included in the pipette solution. They almost completely inhibited the PAR_2_-AP potentiation of *I*_pH 6.6_, compared with an increase of 61.6 ± 4.6% induced by PAR_2_-AP on *I*_pH 6.6_ in normal extracellular solution condition (*P* < 0.01, post hoc Bonferroni’s test, compared with normal internal solution, *n* = 10; Fig. 3c). Although PAR_2_ couples to phospholipase C, leading to stimulation of PKC, PAR2 agonists also increased cAMP generation in DRG neurons and HEK 293 cells, which would activate PKA [37]. H-89, a selective PKA inhibitor, was also applied internally to CHO cells through recording patch pipettes. Pre-application of PAR_2_-AP (10^−5^ M for 1 min) increased *I*_pH 6.6_ to 15.3 ± 5.8% with treatment of 10 μM H-89 (Fig. 3c). These data collectively indicated that the potentiation of ASIC3 currents by PAR_2_-AP was dependent upon GPCR, PLC, PKC, and PKA signaling pathways.

**Fig. 4:**
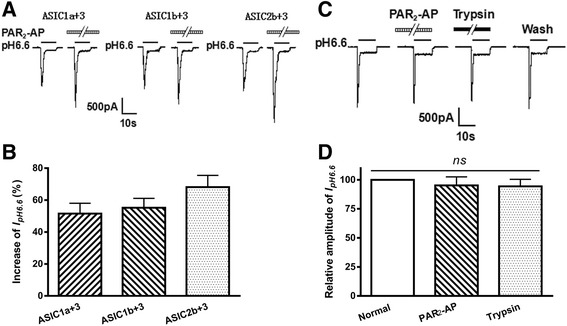
PAR_2_-AP potentiation of proton-gated currents mediated by heteromeric ASIC3 channels. Representative **a** current traces and **b** bar graphs show that *I*_pH 6.6_ was also enhanced by PAR_2_-AP (10^−5^ M) pre-applied for 1 min in CHO cells co-expressing PAR_2_ and heteromeric ASIC3 plus 1a, 1b, 2a, or 2b channels. *n* = 8 in each column. The **c** current traces and **d** bar graphs show that PAR_2_-AP and trypsin had no effect on *I*_pH 6.6_ in CHO cells expressing alone homomeric ASIC3, but not expressing PAR_2_. Currents were normalized to control (100%, *white column*). *n* = 10 in each column

We tested whether PAR_2_-AP could enhance acid-evoked currents mediated by heteromeric channels containing ASIC3. ASIC3-containing heteromeric channels were expressed with PAR_2_ in CHO cells. To minimize the formation of ASIC3 homomers, ASIC3 and another ASIC subunit were co-expressed at the 1:3 ratio in CHO cells. After pretreatment of PAR_2_-AP (10^−5^ M) for 1 min, the peak currents of heteromeric ASIC1a+3, ASIC1b+3, and ASIC2b+3 channels increased 51.6 ± 6.5%, 55.2 ± 5.9%, and 68.1 ± 7.3%, respectively (*n* = 8; Fig. 4a, b). These results show that PAR_2_-AP also enhanced currents induced by the heteromeric ASIC3 channels. We also examined the effects of PAR_2_-AP and trypsin on ASIC3 currents in CHO cells expressing alone ASIC3, but not expressing PAR_2_. Neither PAR_2_-AP nor trypsin had an effect on *I*_pH 6.6_ at a concentration of 10^−5^ M in ASIC3-transfected CHO cells (one-way analysis of variance followed by post hoc Bonferroni’s test, *P* > 0.1, *n* = 10; Fig. 4c, d).

### Potentiation of proton-evoked currents and spikes by the activation of PAR_2_ in rat DRG neurons

**Fig. 5:**
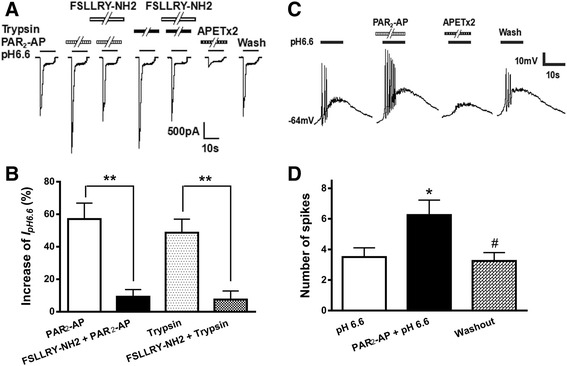
Potentiation of proton-evoked currents and spikes by the activation of PAR_2_ in rat DRG neurons. The **a** current traces and **b** bar graphs show that *I*_pH 6.6_ was enhanced by PAR_2_-AP (10^−5^ M) or trypsin (10^−5^ M) pre-applied alone for 1 min in rat DRG neurons. This enhancing effect of PAR_2_-AP was inhibited by FSLLRY-NH2 (10^−5^ M), a selective PAR_2_ antagonist. Also, this proton-induced current could be completely blocked by 2 μM APETx2, an ASIC3 inhibitor. Currents were evoked by extracellular application of a pH 6.6 solution for 5 s in the presence of capsazepine (10 μM) to block proton-induced TRPV1 activation. DRG neurons with membrane potential clamped at −60 mV. The **c** spike recordings and **d** bar graphs show that pretreatment of PAR_2_-AP (10^−5^ M, for 1 min) increased the acidosis-induced number of action potentials in DRG neurons. The spikes were not evoked by pH 6.6 acidic solution in the presence of 2 μM APETx2. Action potentials were evoked by pH 6.6 acidic solution for 5 s with current clamp recording in the presence of capsazepine (10 μM) to block proton-induced TRPV1 activation. The acidosis-evoked action potentials recovered to control condition after washout of PAR_2_-AP. *P < 0.05, paired *t* test, compared with pH 6.6 column alone; #*P* < 0.05, paired *t* test, compared with PAR_2_-AP + pH 6.6 column, *n* = 9 in each column

ASICs expressed in primary sensory neurons respond to local acidosis with membrane depolarization and spikes, which is thought to be the initial trigger for pain sensation [21]. PAR_2_ is also expressed in primary sensory neurons and activated by endogenous proteases [7, 8]. To gain insights into the pathophysiological function of interaction between ASIC3 and PAR_2_, we next observed whether PAR_2_ activation would also sensitize ASIC3 in acutely isolated rat DRG neurons by patch clamp recording. All proton-gated currents were recorded in the presence of capsazepine (10 μM) to block the proton-induced TRPV1 activation [38]. A rapid reduction of extracellular pH from 7.4 to 6.6 for 5 s evoked an inward current (*I*_pH 6.6_) in most native DRG neurons (72.0%, 36/50, from 12 rats). The acidosis-evoked currents were characterized by a large transient peak current followed by fast inactivation and then a small sustained current with no or very slow inactivation. In rat DRG neurons, ASIC3 is mainly present in heterotrimeric channels, which require higher APETx2 concentrations for inhibition [39]. We found that the ASIC currents are also blocked by 2 μM of APETx2 in eight DRG neurons tested (Fig. 5a). Thus, they may be ASIC3-like currents and were mainly observed in the next study. Similar to that observed in CHO cells co-expressing ASIC3 and PAR_2_, the proton-evoked currents were enhanced by the pre-application of PAR_2_-AP in some DRG neurons sensitive to acidic stimuli (Fig. 5a, b). The peak amplitude of *I*_pH 6.6_ increased 57.1 ± 9.8% after pretreatment with PAR_2_-AP (10^−5^ M) for 1 min in nine DRG neurons tested (Fig. 5b). However, the peak amplitude of *I*_pH 6.6_ only increased 9.3 ± 44% when PAR_2_-AP (10^−5^ M) was co-treated with 10^−5^ M FSLLRY-NH2 (*P* < 0.01, compared with PAR_2_-AP alone column, one-way ANOVA followed by post hoc Bonferroni’s test, *n* = 9), suggesting that potentiation of ASIC currents by PAR_2_-AP was blocked by the addition of FSLLRY-NH2, a selective PAR_2_ antagonist, in rat DRG neurons (Fig. 5a, b). Like PAR_2_-AP, trypsin (10^−5^ M) pre-application to the DRG neurons for 1 min also produced an increase of 48.7 ± 8.3% on *I*_pH 6.6_ (Fig. 5a, b). And the potentiation of ASIC currents by trypsin was also inhibited by 10^−5^ M FSLLRY-NH2 in rat DRG neurons (Fig. 5a, b).

To investigate whether the PAR_2_-AP enhancement of ASIC3 relates to increase neuronal excitability, we recorded action potentials (APs or spikes) in DRG neurons in current clamp mode in the presence of capsazepine (10 μM) to block proton-induced TRPV1 activation [38]. As shown in Fig. 5c, a pH drop from 7.4 to 6.6 for 5 s could trigger bursts of APs in a DRG neuron tested. Consistent with the results that PAR_2_-AP potentiated proton-gated currents under voltage clamp conditions, pretreatment of 10^−5^ M PAR_2_-AP for 1 min also increased acidosis-evoked spikes. In the nine DRG neurons tested from six rats, pretreatment of PAR_2_-AP increased the mean number of spikes induced by acidosis from 3.5 ± 0.6 of control condition to 6.3 ± 0.9 (*P* < 0.05, paired *t* test, *n* = 9) (Fig. 5d). After a washout of PAR_2_-AP, the acidosis-evoked spikes recovered to the control condition. In addition, the acidosis-evoked spikes were completely blocked by 2 μM of APETx2, suggesting that ASIC3-containing channels mediated the spikes (Fig. 5c). These results indicated that the activation of PAR_2_ reversibly increased proton-induced membrane excitability of rat DRG neurons.

### Exacerbation of acidosis-induced ASIC3-dependent nocifensive behaviors by PAR_2_-AP in rats

**Fig. 6:**
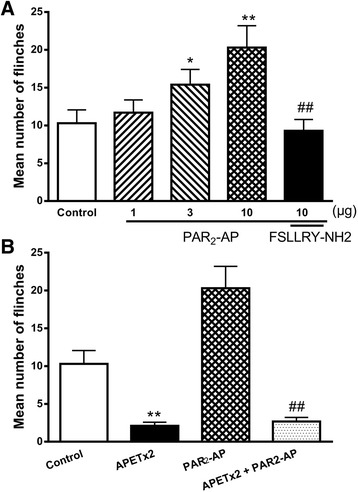
Effect of PAR_2_-AP on nociceptive responses to intraplantar injection of acetic acid in rats. The **a** bar graph shows that the nociceptive responses are evoked by intraplantar injection of acetic acid (30 μl, pH 6.0) in the presence of the TRPV1 inhibitor capsazepine (100 μM). The pretreatment of PAR_2_-AP increased the flinching behavior induced by acetic acid in a dose-dependent manner (1–10 μg). The effect of PAR_2_-AP (10 μg) was blocked by co-treatment of FSLLRY-NH2 (20 μg), a selective PAR_2_ antagonist. *P < 0.05, **P < 0.01, Bonferroni’s post hoc test, compared with control; ##*P* < 0.01, Bonferroni’s post hoc test, compared with PAR_2_-AP (10 μg) column. The **b** bar graph shows that the acidosis-evoked nociception and increased pain response induced by PAR_2_-AP (10 μg) were blocked by pretreatment with APETx2 (20 μl, 20 μM), an ASIC3 inhibitor. **P < 0.01, Bonferroni’s post hoc test, compared with control; ##*P* < 0.01, Bonferroni’s post hoc test, compared with PAR_2_-AP column. Each bar represents the number of flinches that the animals spent licking/lifting the injected paw during first 5-min observation period (mean ± SEM of 10 rats in each group)

The above results demonstrated that ASIC3 activity was potentiated by PAR_2_ activation in vitro. We finally ascertain whether PAR_2_-AP facilitates pain-related behaviors through interacting with ASIC3 in vivo. Acetic acid (0.6%) was injected into the right hind paws of rats and measured the number of flinches that the animals spent licking and/or lifting the injected paw. Intraplantar injection of acetic acid elicits an intense flinch/shaking response which mainly occurred during 0–5 min after injection of acetic acid [21, 32]. We found that pre-administration of PAR_2_-AP dose-dependently exacerbated the acidosis-induced nocifensive behaviors (Fig. 6a). The acetic acid-induced number of flinches was significantly greater in rats pretreated with medium and high dose (3 and 10 μg) of PAR_2_-AP than that observed in rats injected with acetic acid alone (Bonferroni’s post hoc test, *P* < 0.05 and *P* < 0.01, *n* = 10). However, the low dose (1 μg) of PAR_2_-AP had no effect on the acidosis-induced nocifensive behaviors (Bonferroni’s post hoc test, *P* > 0.1, *n* = 10). In addition, the exacerbating effect of 10 μg PAR_2_-AP on acidosis-induced nocifensive behaviors was blocked by co-administration of 20 μg FSLLRY-NH2, a selective PAR_2_ antagonist (Bonferroni’s post hoc test, *P* < 0.01, compared with 10 μg PAR_2_-AP alone, *n* = 10; Fig. 6a). These results indicated that periphery activation of PAR_2_ by PAR_2_-AP contributed to acidosis-induced nocifensive behaviors in rats. Acetic acid-induced nociceptive response in rats was potently blocked by treatment with APETx2 (20 μM, 20 μl), an ASIC3 blocker, demonstrating the involvement of ASIC3 in the acidosis-induced nociception (Fig. 6b). In addition, the increased ASIC3-mediated pain behavior induced by 10 μg PAR_2_-AP can also be potently inhibited by treatment with APETx2 (20 μM, 20 μl; Fig. 6b).

## Discussion

We found that there was a functional interaction between PAR_2_ and ASIC3 in transfected cell lines, DRG neurons, and intact animals. The present study provided electrophysiological and behavioral evidences that activation of PAR_2_ can sensitize ASIC3.

In CHO cells expressing ASIC3 and PAR_2_ and rat DRG neurons, a rapid drop in the extracellular pH from 7.4 to 6.6 evoked an inward current that can be characterized by a large transient current followed by fast inactivation and then a small sustained current with no or very slow inactivation [33]. These acidosis currents were mediated by ASIC3-containing homomeric and heteromeric channels, since peak currents could be blocked by APETx2, an ASIC3 blocker, although it also inhibits voltage-gated Na^+^ channels at higher concentration [40]. In peripheral sensory neurons, ASIC3 is detected in axons, axon terminals, and cell bodies, where its activation contributes to pain signaling [20–22]. ASIC3 has emerged as critical pH sensors predominantly expressed in nociceptors [22]. We found that activation of PAR_2_ by PAR_2_-AP produced an enhancing effect on ASIC3 currents in CHO cells transfected with homomeric and heteromeric ASIC3 and PAR_2_. PAR_2_-AP sensitized ASIC3 by increasing the maximum response without changing the EC_50_ values. Trypsin, a possible physiological ligand of the PAR_2_, had a similar potentiating effect on ASIC3 currents. PAR_2_-AP and trypsin increased ASIC3 and ASIC3-like currents through PAR_2_, since their effects were blocked by FSLLRY-NH2, a selective PAR_2_ antagonist, in transfected CHO cells and DRG neurons. However, neither PAR_2_-AP nor trypsin had an effect on ASIC3 currents in CHO cells expressing alone ASIC3, but not expressing PAR_2_. These results indicated that a functional interaction occurred between PAR_2_ and ASIC3.

The current study showed that PAR_2_-AP potentiation of ASIC3 currents was blocked by intracellular dialysis of GDP-β-S, indicating that G proteins were involved in the intracellular mechanisms of this potentiation. PAR_2_ primarily couple the G_q/11_ subtype of G protein family, which activates PLC [1]. Lack of the potentiating effect in cells treated with PLC inhibitor U-73122 indicated a PLC-dependent pathway is predominantly involved in functional interaction between PAR_2_ and ASIC3. One of the consequences of PLC activation is the breakdown of PIP2 into DAG and inositol triphosphate, followed by mobilization of calcium and activation of PKC. Our observation that PKC inhibitor GF109203X also prevented the potentiation of ASIC3 currents by PAR_2_-AP indicated that activation of PKC played a major role in PAR_2_-induced sensitization of ASIC3. Similarly, electrophysiological studies have suggested that PAR_2_ sensitizes TRPV1, TRPV4, and TRPA1, which was blocked by a PLC inhibitor [14, 16, 18]. It has been shown that ASIC3 is modulated by proinflammatory mediators such as serotonin and bradykinin via a PKC pathway [41–43]. We recently reported that G_q/11_-coupled metabotropic receptor activation such as glutamate (mGluRs), ATP (P2Y), and serotonin (5-HT_2_) receptors causes potentiation of ASICs in a PKC-dependent manner in rat DRG neurons [34–36]. PAR_2_ has been found to sensitize TRPV1, TRPV4, and P2X3 ATP receptor in a PKC- and PKA-dependent manner [16, 37, 44]. Our observation that inhibition of PKA with H-89 reduced the potentiation of ASIC3 currents by PAR_2_-AP indicated that PKA also mediated PAR2-induced sensitization of ASIC3. It has been shown that heteromeric ASIC3/ASIC2b channels, but not homomeric ASIC3 channels, are regulated by PKC and this regulation requires PICK1 [42]. The present study showed that PAR2-induced sensitization of homomeric ASIC3 channels required simultaneous activation of PKC and PKA, since blocking either of the these kinases prevented the potentiation of ASIC3 currents by PAR_2_-AP. It remains to be determined whether these kinases act sequentially or in parallel.

The present study showed that PAR_2_-AP potentiated acidosis-evoked currents and membrane excitability in dissociated rat DRG neurons, indicating that PAR_2_ activation also sensitized ASIC3 in rat sensory neurons. In consistent with our previous report [34–36], a rapid reduction of extracellular pH evoked an ASIC3 current in most native DRG neurons, since the proton-evoked currents were blocked by APETx2. Similar to that observed in CHO cells co-expressing ASIC3 and PAR_2_, pre-application of PAR_2_-AP or trypsin also enhanced the proton-evoked currents through PAR_2_ in some DRG neurons sensitive to acidic stimuli. Recently, it has been reported that the activation of PAR_2_ enhances weak acid-induced ATP release through the sensitization of TRPV1 and ASIC3 in human esophageal epithelial cells [45]. Extracellular acidic stimuli open ASICs and mainly induce sodium influx, which can depolarize membrane potentials to the threshold of excitability and result in bursts of action potentials. The current study showed that acidosis-evoked action potentials were enhanced by PAR_2_-AP. The increased acidosis-evoked neuronal excitability appeared to correlate with PAR_2_-AP potentiation of ASIC3 currents in voltage clamp experiments. Moreover, pain sensation that was caused through the ASIC3 was also potentiated by the PAR_2_ activation. In the behavior studies, we found that intraplantar pretreatment of PAR_2_-AP dose-dependently exacerbated the acidosis-induced nocifensive behaviors in rats. The combined data indicated that PAR_2_ activation indeed increased ASIC3 activity, not only at the cellular level but also at the behavioral level.

ASIC3 is expressed in both DRG cell bodies and sensory terminals, which monitors extracellular pH fall and contributes to proton-evoked pain signaling [20, 21]. It has been shown that ASIC3 plays an important role in various pain conditions such as inflammatory pain, postoperative pain, and migraine [22, 29, 31]. PAR_2_ is also expressed on a subset of primary sensory neurons and functionally involved in peripheral mechanisms of inflammation and pain [7, 8]. Activation of PAR_2_ on sensory nerve ending evokes thermal and mechanical hyperalgesia [9]. Our observation that PAR_2_ activation sensitized ASIC3 is likely to be of physiological relevance in pathological condition. For example, ASIC3 plays an important role in postoperative pain, while PAR2 activation by mast cell tryptase is involved in postoperative pain [12, 29]. Protons are released from damaged cells and the de-granulation of mast cells during tissue injury and inflammation, and extracellular pH values can drop to 5.4 [25, 26, 46]. Trypsin and tryptase, the selective agonists on physiological state for PAR_2_, could be released from different cell types including mast cells in peripheral tissue and visceral organs during tissue injury and inflammation [2, 47, 48]. The endogenous proteases can activate PAR_2_ expressed in peripheral neuronal terminals. As a GPCR, PAR_2_ activation itself may be not sufficient to induce action potentials in primary afferents [15]. Thus, the underlying mechanism of PAR_2_-mediated hyperalgesia may involve the interaction between PAR_2_ and other molecules such as ion channels. During inflammation and injury, it is possible that both proteases and protons release together. The released protons are sufficient to activate ASIC3, subsequently evoke action potentials, and produce pain signaling in primary afferents [26]. Proteases cleave and activate PAR_2_ in peripheral sensory terminals. PAR_2_ subsequently activates G proteins, which result in PKC activation via PLC and PKA. The current study demonstrated that the PAR_2_ signaling may further sensitize ASIC3 in nociceptors, which exacerbated acidosis-evoked nociception.

## Conclusions

We have revealed a functional interaction between PAR_2_ and ASIC3. Activation of PAR_2_ signaling sensitized ASIC3 in a combination of observations in transfected cell lines, DRG, and intact animals. Sensitization of ASIC3 by PAR_2_ required activation of PLC, PKC, and PKA, which contributed to acidosis-evoked pain. Our results indicated a novel peripheral mechanism underlying PAR_2_ involvement in hyperalgesia by sensitizing ASIC3 in primary sensory neurons. Targeting one or more of these signaling molecules may present new opportunities for the treatment of acidosis-mediated pain.

## Abbreviations

ASICs: Acid-sensing ion channels
CHO: Chinese hamster ovary
DRG: Dorsal root ganglion
EC_50_: Half-maximal response
GPCRs: G protein-coupled receptors
*I*_pH_: Proton-gated current
PAR_2_-AP: PAR_2_-activating peptide
PARs: Proteinase-activated receptors
TRP: Transient receptor potential
